# Estimation of glomerular filtration rate by a radial basis function neural network in patients with type-2 diabetes mellitus

**DOI:** 10.1186/1471-2369-14-181

**Published:** 2013-08-29

**Authors:** Xun Liu, Yan-Ru Chen, Ning-shan Li, Cheng Wang, Lin-Sheng Lv, Ming Li, Xiao-Ming Wu, Tan-Qi Lou

**Affiliations:** 1 Division of Nephrology, Department of Internal Medicine, The Third Affiliated Hospital of Sun Yat-sen University, Guangzhou, China; 2 College of Biology Engineering, South China University of Technology, Guangzhou, China; 3 Department of Radiation Oncology, Chengdu International Cancer Treatment Hospital, Chengdu, China; 4 Operating Room, The Third Affiliated Hospital of Sun Yat-sen University, Guangzhou, China

**Keywords:** Type 2 diabetes, Chronic kidney disease, Glomerular filtration rate, Artificial neural network

## Abstract

**Background:**

Accurate and precise estimates of glomerular filtration rate (GFR) are essential for clinical assessments, and many methods of estimation are available. We developed a radial basis function (RBF) network and assessed the performance of this method in the estimation of the GFRs of 207 patients with type-2 diabetes and CKD.

**Methods:**

Standard GFR (sGFR) was determined by ^99m^Tc-DTPA renal dynamic imaging and GFR was also estimated by the 6-variable MDRD equation and the 4-variable MDRD equation.

**Results:**

Bland-Altman analysis indicated that estimates from the RBF network were more precise than those from the other two methods for some groups of patients. However, the median difference of RBF network estimates from sGFR was greater than those from the other two estimates, indicating greater bias. For patients with stage I/II CKD, the median absolute difference of the RBF network estimate from sGFR was significantly lower, and the P_50_ of the RBF network estimate (n = 56, 87.5%) was significantly higher than that of the MDRD-4 estimate (n = 49, 76.6%) (*p* < 0.0167), indicating that the RBF network estimate provided greater accuracy for these patients.

**Conclusions:**

In patients with type-2 diabetes mellitus, estimation of GFR by our RBF network provided better precision and accuracy for some groups of patients than the estimation by the traditional MDRD equations. However, the RBF network estimates of GFR tended to have greater bias and higher than those indicated by sGFR determined by ^99m^Tc-DTPA renal dynamic imaging.

## Background

Diabetic nephropathy is the leading cause of end stage renal disease, a condition characterized by abnormal glomerular filtration rate (GFR) and serum creatinine (SCr) [1]. The National Kidney Foundation (NKF) considers GFR as the best overall measure of kidney function in healthy and diseased individuals [2]. However, measurement of GFR by use of radioisotopes is time-consuming and expensive, so this method is not used in routine clinical practice. Instead, numerous equations have been proposed to estimate the GFR without the need for radioisotopes [2]. These equations consider SCr and several additional variables, such as age, gender, race, and body size [2]. The American Diabetes Association [3] also recommends estimation of GFR from serum creatinine (SCr) -based formulae, such as a Modification of Diet in Renal Disease (MDRD) study equation [4].

However, these equations may yield inaccurate estimates in some populations, such as elderly Chinese patients with CKD [5]. Recent studies have criticized the equations currently used to estimate GFR in diabetic patients [6-10]. In particular, the Chronic Kidney Disease Epidemiology Collaboration (CKD-EPI) equation [6], the Mayo Clinic Quadratic (MCQ) equation [7], and the four-variable MDRD equation [8] all underestimated GFR in patients with type-2 diabetes, and the Cockcroft-Gault equation overestimated GFR in patients with type-2 diabetes [10]. These equations may be inaccurate because they do not account for ethnicity [11]. For example, in a group of Chinese patients with CKD, the MDRD equation 7 and the abbreviated MDRD equations underestimated GFR in patients with near-normal renal function and overestimated GFR in patients with advanced renal failure [12]. These equations may also be inaccurate because they were developed by linear regression methods [11,13,14]. Linear regression models do not account for the non-linear physiological processes that underlie GFR. Thus, it is important to develop better methodologies for estimation of GFR.

Artificial neural networks (ANNs) have been successfully used to model non-linear phenomena in the field of engineering forecasting. Modern ANNs provide effective nonlinear mapping of data, good fault tolerance, and good self-organization [15,16]. Previous research demonstrated that an ANN was more accurate than a logistic regression model in prediction of clinical outcome in patients with systemic inflammatory response syndrome and hemodynamic shock [17]. Other research groups have used ANNs to estimate GFR, including a knowledge-based neural network (KBNN) model [18], an evolving connectionist systems (ECOS) model [19], and a tree-based model with 6 terminal nodes [15]. In all of these cases, the ANNs provided better estimates of GFR than the traditional equations.

Radial basis function (RBF) networks are among the most widely used ANNs, but there have been limited clinical applications of these networks. Our previous study [20] described a simple RBF network for estimation of GFR (eGFR_RBF_) in a group of 327 Chinese patients with chronic kidney disease (CKD). The results indicated that the eGFR_RBF_ had less bias and greater precision than the traditional MDRD equations. The accuracy (deviation less than 30% from the sGFR) of the eGFR_RBF_ was significantly better than those from traditional eGFR equations, such as the Jelliffe-1973-equation and the Ruijin-equation [20].

In the present study, we tested the precision and accuracy of an RBF network model for estimation of GFR in an independent group of 207 Chinese patients who had type-2 diabetes and CKD and compared the results of the RBF network method with the results from two traditional MDRD formulae [13].

## Methods

### Patients

From January 2005 through December 2009, 207 consecutive patients with type-2 diabetes from the Third Affiliated Hospital of Sun Yat-sen University (Guangzhou, China) were enrolled. Patients younger than 18 years, taking cimetidine or trimethoprim, with acute kidney deterioration, clinical edema, skeletal muscle atrophy, pleural effusion or ascites, malnutrition, amputation, heart failure, or ketoacidosis were excluded. None of the patients were treated by dialysis during the study. CKD was staged according to the National Kidney Foundation (NKF) – Kidney Disease Outcomes Quality Initiative clinical practice guidelines [2] based on the GFR measured by ^99m^Tc-DTPA dynamic imaging method. Patients were placed into 3 groups based on CKD stage: *(i)* Stage I/II CKD (GFR ≥ 60 mL/min/1.73 m^2^); *(ii)* Stage III CKD (GFR = 30–59 mL/min/1.73 m^2^); or *(iii)* Stage IV/V CKD (GFR < 30 mL/min/1.73 m^2^). The study protocol was approved by the institutional review board at the Third Affiliated Hospital of Sun Yat-sen University and all patients provided informed consent. All participants provided written informed consent.

### Measurements

GFR measured by the ^99m^Tc-DTPA renal dynamic imaging method (modified Gate’s method) was used as the standard GFR (sGFR) [21,22], and was calculated as described by Li et al. [23]. The gamma camera uptake method with ^99m^Tc-DTPA is a simple method for determination of GFR, and is less time-consuming, and less expensive than other methods [24]. Moreover, this method has been recommended as the reference approach for determination of GFR by the Nephrology Committee of Society of Nuclear Medicine [25], and is widely used as a standard method for evaluation of kidney function and estimation of GFR in China. ^99m^Tc-DTPA renal dynamic imaging was measured by a Millennium TMMPR SPECT using the General Electric Medical System, as described previously [22]. Serum albumin (Alb) and blood urea nitrogen (BUN) were assayed on a Hitachi 7180 autoanalyzer (Hitachi, Tokyo, Japan; reagents from Roche Diagnostics, Mannheim, Germany). SCr was measured by an enzymatic method on the same instrument according to the manufacturer’s instructions. SRM967 (standard reference material released by NIST for serum creatinine calibration) was used for calibration. Patient sex, age, height, and weight were recorded at the same time.

### RBF network

An ANN is a computational method composed of interconnected artificial neurons (mathematical functions) that processes information and that typically consists of an input layer, one or more hidden layers, and an output layer. ANNs are used in diverse scientific and engineering fields to model the complex relationships of inputs and outputs. An RBF network is a feed-forward network with one hidden layer, in which activation of the hidden layer is a nonlinear radial basis function (a function whose value only depends on the distance to the origin).

In this study, the input layer consisted of measured serum creatinine (SCr) and the output layer consisted of sGFR. Our previous work [20] indicated that when SCr was measured by the enzymatic method, a simple RBF network model successfully estimated GFR in a population of 327 Chinese patients with CKD, based on analysis of all patients and on analysis of subgroups of patients with different stages of CKD [20]. In this previous study, the RBF network was a feed-forward ANN with an input layer of one unit (SCr), one hidden layer, and an output layer of one unit (sGFR), which was measured in all 327 CKD patients. This RBF network was constructed by use of the newrbe function in MathWorks. In the present study, we tested this RBF network in an independent group of 207 patients who had type-2 diabetes (external validation data set) to verify the original results.

### MDRD equations

GFR was also estimated by the traditional MDRD equations [13]. In particular, we used the re-expressed 4-variable MDRD equation (R-MDRD4):

$$\begin{array}{c} {\mathrm{eGFR}}_{4}=175\times {\mathrm{Scr}}^{‒1.154}\times {\mathrm{Age}}^{‒0.203}\\ \;\times \left[0.742\;\mathrm{if}\;\mathrm{female}\right]\times \left[1.212\;\mathrm{if}\;\mathrm{black}\right] \end{array}$$

and the re-expressed 6-variable MDRD equation (R-MDRD6):

$$\begin{array}{c} {\mathrm{eGFR}}_{6}=161.5\times {\mathrm{Scr}}^{‒0.999}\times {\mathrm{Age}}^{‒0.176}\times {\mathrm{BUN}}^{‒0.17}\\ \;\times {\mathrm{Alb}}^{+0.318}\times \left[0.762\;\mathrm{if}\;\mathrm{female}\right]\\ \;\times \left[1.18\;\mathrm{if}\;\mathrm{black}\right] \end{array}$$

### Statistical analysis

All demographic and clinical data are summarized as means ± standard deviations (SDs), as medians and inter-quartile ranges (IQRs: Q1, Q3) for continuous variables that had non-normal distributions, and as N and percent for categorical data (CKD stage). Data were compared using a one-way ANOVA with Bonferroni’s *post hoc* correction, the Kruskall Wallis test, the Mann–Whitney U test for pair-wise comparisons of data that had non-normal distributions, or Pearson’s Chi-square test (gender).

The overall differences between eGFR and sGFR are summarized as medians and IQRs due to the non-normal distributions. Differences among patients with different CKD stages were compared with the Kruskall Wallis test with a *post hoc* method or with the Mann–Whitney U test for pair-wise comparisons. Within-group comparisons of measurements were performed using the Wilcoxon signed ranks test for a given CKD stage.

The accuracy of eGFR are summarized as N and percent of patients with eGFR differing less than 15% (P_15_), 30% (P_30_), and 50% (P_50_) from sGFR. Accuracy of the estimates was compared for patients with different CKD stages using the Pearson Chi-square test. The accuracies of eGFR values were compared using the McNemar test within the same CKD stage. Bland-Altman plots (eGFR_4_*vs.* sGFR, eGFR_6_*vs.* sGFR, and eGFR_RBF_*vs.* sGFR) were graphed with Medcalc for Windows (ver. 9.3.9.0, Mariekerke, Belgium). The 3 different methods of estimating GFR were also used to classify patients by CKD stage. A Wilcoxon sign-rank test was used to compare the differences of CKD stages from sGFR and each of these estimates.

All statistical assessments were two-tailed and a *p*-value less than 0.05 was considered significant. The significance level was adjusted by Bonferroni’s method to 0.0167 (0.05/3) and 0.01 (0.05/4) for *post hoc* pair-wise comparisons of CKD stages and eGFR, respectively. All statistical analysis was performed using SPSS (version 11.0 SPSS, Chicago IL, USA).

## Results

A total of 207 patients (119 males, 88 females) with a mean age of 61.43 years (SD = 12.03) were enrolled. Table 1 shows the baseline demographic and clinical characteristics of the 207 patients and of sub-groups with different stages of CKD. There were 64 patients (30.9%) with stage I/II disease, 81 patients (39.1%) with stage III disease, and 62 patients (30%) with stage IV/V disease. As expected, patients with more severe disease had lower serum Alb and sGFR, and higher SCr and BUN (Table 1).

**Table 1:** Demographic and clinical characteristics of patients with type-2 diabetes and different stages of CKD (n = 207)

**Variable**	**Total**^**1**^**(n = 207)**	**Stage I/II**^**1**^**(n = 64)**	**Stage III**^**1**^**(n = 81)**	**Stage IV/V**^**1**^**(n = 62)**	**P-value**
Age (years)	61.43 ± 12.03	56.64 ± 11.65	62.78 ± 11.53^a^	64.61 ± 11.72^a^	<0.001^*^
Sex					0.177
Males	119 (57.5)	34 (53.1)	53 (65.4)	32 (51.6)	
Females	88 (42.5)	30 (46.9)	28 (34.6)	30 (48.4)	
BMI (kg/m^2^)	23.42 (21.45 , 25.83)	23.54 (21.32 , 25.77)	22.86 (21.16 , 25.52)	24.05 (22.04 , 25.92)	0.262
BSA (m^2^)	1.66 (1.54 , 1.80)	1.66 (1.52 , 1.77)	1.64 (1.54 , 1.81)	1.69 (1.54 , 1.77)	0.826
Alb (g/dL)	3.75 (3.19 , 4.20)	4.00 (3.51 , 4.41)	3.83 (3.30 , 4.18)	3.39 (2.99 , 3.84)^ab^	<0.001^*^
SCr (mg/dL)	1.52 (0.92 , 3.72)	0.77 (0.57 , 1.09)	1.58 (1.09 , 2.39) ^a^	5.19 (2.99 , 7.01)^ab^	<0.001^*^
BUN (mg/dL)	27.17 (17.76 , 48.54)	16.70 (13.74 , 21.25)	27.90 (18.74 , 44.82)^a^	54.68 (41.29 , 70.60)^ab^	<0.001^*^
DTPA-GFR (mL/min/1.73 m^2^)	43.45 (25.35 , 64.36)	78.39 (66.86 , 87.89)	43.42 (35.81 , 54.82) ^a^	19.70 (14.58 , 23.19)^ab^	<0.001^*^
GFR ≧60 mL/min/1.73 m^2^	64 (30.9)	64 (100)	0 (0)	0 (0)	
GFR <60 mL/min/1.73 m^2^	143 (69.1)	0 (0)	81 (100)	62 (100)	

Abbreviations: *BMI* body mass index, *BSA* body surface area, *Alb* albumin, *SCr* serum creatinine, *BUN* blood urea nitrogen, *DTPA-GFR* GFR measured by ^99m^Tc-DTPA renal dynamic imaging.

^1^ Continuous data are given as means ± SDs for age, as medians and IQRs for continuous variables with non-normal distributions, and as n (%) for categorical variables. Differences among patients with different stages of CKD were compared with a one-way ANOVA and a Bonferroni *post-hoc* comparison for age, a Kruskall Wallis test with Mann–Whitney U test for pair-wise comparison of other not normally distributed continuous data; and Pearson’s Chi-square test for sex.

*p < 0.05, indicates significant difference among CKD stages.

^a,b^*p* < 0.0167 (0.05/3), indicates significant difference of CKD stage I/II and III, respectively.

Table 2 shows the sGFR values and the three different estimates of GFR (eGFR_4_, eGFR_6_, and eGFR_RBF_) for all 207 patients and for sub-groups with different stages of CKD. As expected, GFR values were lower in patients with more advanced disease. The eGFR_RBF_ of all 207 CKD patients and of patients with different stages of CKD were significantly higher than the sGFR values (*p* < 0.01 for all). In addition, for patients with stage III or stage IV/V CKD, the eGFR_RBF_ values were significantly higher than those from eGFR_4_ and eGFR_6_ (*p* < 0.01 for both).

**Table 2:** **Glomerular filtration rates (sGFR, eGFR**_**4**_**, eGFR**_**6**_**, and eGFR**_**RBF**_**) of patients with different stages of CKD**

**Measurement method (mL/min/1.73 m**^**2**^**)**	**Total**^**1**^**(n = 207)**	**Stage I/II**^**1**^**(n = 64)**	**Stage III**^**1**^**(n = 81)**	**Stage IV/V**^**1**^**(n = 62)**	**P-value**
sGFR	43.45 (25.35 , 64.36)	78.39 (66.86 , 87.89)	43.42 (35.81 , 54.82)^a^	19.70 (14.58 , 23.19)^ab^	<0.001^*^
eGFR_4_	42.43 (15.68 , 75.46)	89.12 (67.73 , 124.20)^†^	42.43 (26.50 , 59.31)^a^	9.60 (6.71 , 17.11)^ab†^	<0.001^*^
eGFR_6_	39.98 (15.28 , 72.28)^‡^	90.88 (64.52 , 116.84)^‡^	40.21 (24.72 , 60.54)^a‡^	9.92 (6.54 , 17.02)^ab†^	<0.001^*^
eGFR_RBF_	52.25 (34.21 , 81.64)^†‡§^	94.23 (70.34 , 115.27)^†^	50.42 (39.61 , 70.57)^a†‡§^	24.01 (18.70 , 37.28)^ab†‡§^	<0.001^*^

Abbreviations here and in Tables 3: *sGFR* standard GFR, *eGFR*_*4*_ GFR estimated by re-expressed MDRD-4, *eGFR*_*6*_ GFR estimated by re-expressed MDRD-6, *eGFR*_*RBF*_ GFR estimated by RBF network.

^1^ Data are given as medians and IQRs for continuous variables because of non-normal distributions; differences among CKD stages were compared using the Kruskall Wallis test with a *post hoc* method, or the Mann–Whitney U test for pair-wise comparisons; within-group comparisons of the four measurements were performed using Wilcoxon Signed Ranks Test for a given CKD stage.

*p < 0.05, indicates significant difference among CKD stages.

^a,b^*p* < 0.0167 (0.05/3), indicates significant difference in a comparison of CKD stage I/II and III, respectively.

^†,‡,§^, *p* < 0.01 (0.05/4), indicates significant difference in a comparison with sGFR, eGFR_4_, and eGFR_6_, respectively.

The validity of an estimate is a function of its accuracy and precision, and a valid estimate should be close to the true value (low bias) and be reproducible [26]. Table 3 presents the overall performances of the 3 different estimates of GFR in all 207 patients and in patients with different stages of CKD. Each row in the table shows the median difference of eGFR and sGFR and the inter-quartile range (IQR), the median absolute difference of eGFR and sGFR and the IQR, and the percent of GFR estimates within 15% (P_15_), 30% (P_30_), and 50% (P_50_) of sGFR [26]. The results indicate that for all 207 patients, the IQR of the eGFR_RBF_ was smaller than those from eGFR_4_ and eGFR_6_, indicating better precision for the RBF network; however, the median difference of the eGFR_RBF_ from sGFR was significantly higher than those from eGFR_4_ and eGFR_6_ (*p* < 0.0167 for both). The same trends occurred in patients with stage III CKD and stage IV/V CKD. The eGFR_RBF_ had a lower median absolute difference from sGFR and a higher percent of estimates within 15% (P_15_) and 30% (P_30_) of sGFR, suggesting that eGFR_RBF_ had better accuracy. However, the differences between eGFR_RBF_ and eGFR_4_ and eGFR_6_ were not statistically different. The results also indicate that for the patients with stage I/II CKD, the median absolute difference of eGFR_RBF_ and sGFR (19.73) was significantly less than that of eGFR_4_ and sGFR (27.28) (*p* < 0.0167), although the P_50_ for eGFR_RBF_ (n = 56, 87.5%) was significantly higher than that for eGFR_4_ (n = 49, 76.6%) (*p* < 0.0167).

**Table 3:** Overall performance of different methods used to estimate glomerular filtration rate in patients with different stages of CKD

	**Median difference of eGFR and sGFR**^**2**^	**Median absolute difference of eGFR** **and sGFR**^**2**^	**15% (P**_**15**_**)**	**30% (P**_**30**_**)**	**50% (P**_**50**_**)**
Total (n = 207)					
eGFR_4_ and sGFR^1^	-2.83 (-11.23 , 12.13)	32.54 (15.74 , 58.97)	49 (23.7%)	92 (44.4%)	140 (67.6%)
eGFR_6_ and sGFR^1^	-4.06 (-12.59 , 11.21)†	32.78 (16.43 , 57.38)	49 (23.7%)	91 (44.0%)	139 (67.1%)
eGFR_RBF_ and sGFR	9.76 (-0.19 , 18.02)†‡	26.24 (13.29 , 55.82)	62 (30.0%)	112 (54.1%)	149 (72.0%)
Stage I/II (n = 64)					
eGFR_4_ and sGFR^1^	9.23 (-9.20 , 37.35)	26.41 (12.20 , 49.65)	19 (29.7%)	35 (54.7%)	49 (76.6%)
eGFR_6_ and sGFR^1^	4.13 (-14.66 , 32.62)†	27.28 (12.73 , 44.42)	16 (25.0%)	33 (51.6%)	49 (76.6%)
eGFR_RBF_ and sGFR	12.03 (-4.98 , 27.83)	19.73 (12.98 , 35.56)†	21 (32.8%)	43 (67.2%)	56 (87.5%)†
Stage III (n = 81)					
eGFR_4_ and sGFR^1^	-1.04 (-12.85 , 9.92)	27.01 (13.76 , 58.47)	22 (27.2%)	43 (53.1%)	59 (72.8%)
eGFR_6_ and sGFR^1^	-4.80 (-14.07 , 8.97)†	29.82 (13.65 , 57.57)	25 (30.9%)	41 (50.6%)	58 (71.6%)
eGFR_RBF_ and sGFR	7.96 (-1.22 , 21.47)†‡	25.40 (11.23 , 48.50)	26 (32.1%)	47 (58.0%)	61 (75.3%)
Stage IV/V (n = 62)					
eGFR_4_ and sGFR^1^	-6.48 (-11.06 , -0.21)	46.90 (30.81 , 64.90)	8 (12.9%)	14 (22.6%)	32 (51.6%)
eGFR_6_ and sGFR^1^	-6.99 (-11.23 , -1.63)	46.49 (26.56 , 65.17)	8 (12.9%)	17 (27.4%)	32 (51.6%)
eGFR_RBF_ and sGFR	8.89 (1.95 , 15.48)†‡	49.20 (14.98 , 89.37)	15 (24.2%)	22 (35.5%)	32 (51.6%)

^1^ Abbreviations as in Table 2.

^2^ Data are given as median difference (IQRs) and median absolute difference (IQRs) between eGFR and three types of eGFR. Within-group comparisons between the measurements were performed using the Wilcoxon Signed Ranks Test for a given CKD stage.

^3^ The accuracy of eGFR was expressed as n (%) of measurements within 15% (P_15_), 30% (P_30_), and 50% (P_50_) of sGFR. Data within-measurements were compared using the McNemar test for a given CKD stage.

^*^p < 0.05, indicates significant difference among CKD stages.

^†,‡^, P < 0.0167 (0.05/3), indicates significant difference with eGFR_4_ and sGFR and eGFR_6_ and sGFR, respectively.

**Figure 1:**
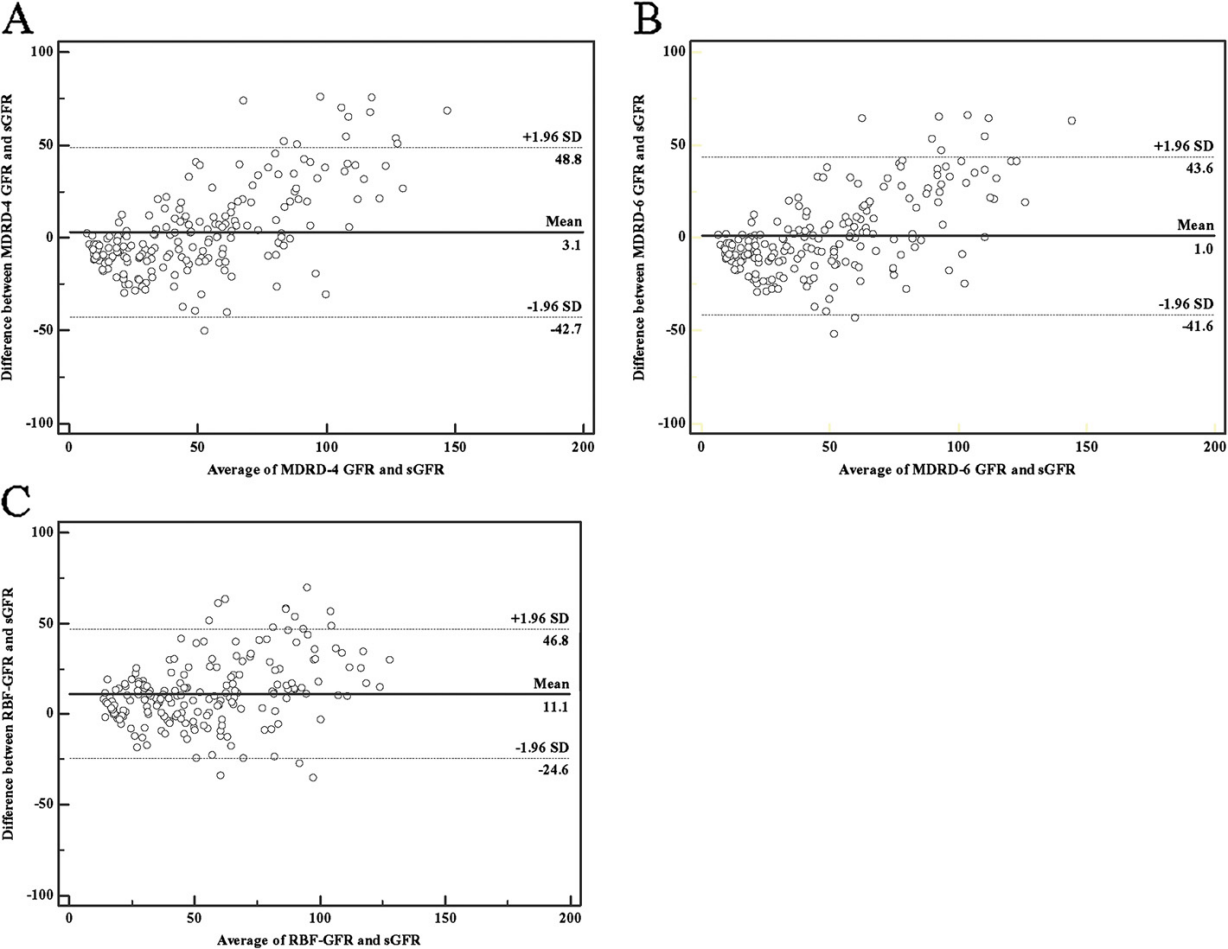
**Bland-Altman plots of the estimation of GFR (mL/min/1.73 m**^**2**^**) relative to sGFR by the (A) re-expressed 4-variable MDRD equation (eGFR**_**4**_**), (B) re-expressed 6-variable MDRD equation (eGFR**_**6**_**), and (C) RBF network (eGFR**_**RBF**_**).** Solid lines represent mean differences and dashed lines represent 95% limits of agreement of the mean difference.

Figure 1 shows Bland-Altman plots for comparisons of sGFR with GFR estimated by eGFR_4_ (A), eGFR_6_ (B), and eGFR_RBF_ (C). The precision is indicated by the distance between the dashed lines (95% limits of agreement) [27]. The results indicate that the distance between lines of 95% limits of agreement was 71.4 for eGFR_RBF_, significantly lower than that for eGFR_4_ (91.5) and eGFR_6_ (85.2). Thus, this analysis also indicates that the eGFR_RBF_ had better precision than the two other estimates from the MDRD equations. However, Figure 1 also shows that the mean differences of sGFR and eGFR_4_ was 3.1, sGFR and eGFR_6_ was 1.0, and sGFR and eGFR_RBF_ was 11.1. This indicates that eGFR_RBF_ had greater bias than the estimates from the MDRD equations and over-estimate the GFR.

## Discussion

We compared the performance of an RBF neural network in the estimation of GFR with the performance of two traditional GFR estimates based on the MDRD equations (MDRD-4 and MDRD-6) in patients with type-2 diabetes and different stages of CKD. Our results indicate that the RBF network provided more precise estimates of GFR than the MDRD equations, and also provided significantly more accurate estimates of GFR for patients with stage I/II CKD. However, the RBF network also had higher bias than the traditional MDRD equations. In particular, the eGFR_RBF_ tended to over-estimate GFR more than eGFR_4_ and eGFR_6_, especially for patients with CKD stage IV/V (Table 2).

In the field of medical data processing, the theoretical basis for the use of statistical regression methods is the “law of large numbers”. That is, the difference of the average of many measurements from the true value should be smaller as more measurements are recorded. However, application of a model derived from one data set to another data set may yield poorer accuracy. Moreover, regression methods can only be used for a limited number of models, and interactions among variables places limits on their use. ANNs have no *a priori* requirement for data distribution, and can handle multi-collinear input variables, neither of which can be managed by regression methods.

These advantages of ANNs have led to their use in several previous studies for estimation of GFR. Song et al. [18] used a knowledge-based neural network model (KBNN) for evaluation of renal function based on 441 GFR data vectors from 141 patients. Their proposed GFR prediction model had at least 10% better accuracy than any of the individual regression formulae or a standard neural network model. Marshall et al. [19] used evolving connectionist systems (ECOS), in which computing structures are trained to generate output from a given set of input variables. They concluded that their ECOS model provided better prediction of GFR in routine clinical practice. No ANNs have been used to estimate GFR of patients with type-2 diabetes mellitus.

It is noteworthy that the RBF network used in this study was only based on SCr measurements, in contrast to the re-expressed MDRD equations, which require measurement of 4 or 6 variables. The NKF does not recommend use of SCr alone for assessment of kidney function [2]. However, previous research indicated that use of fewer variables can yield acceptable estimates of GFR. For example, Bevc et al. [28] reported that a cystatin C-based estimate, which only requires measurement of serum cystatin C, is a reliable marker of GFR in elderly patients and is comparable to the creatinine-based formulae, including the CKD-EPI formulae. Our results suggest that an RBF model based on a single measurement (SCr) can provide precise and accurate estimates of GFR.

There are several limitations to our study. First, SCr was measured by the enzymatic method. Peake et al. [29] indicated that the enzymatic creatinine assay, although theoretically more specific, can have interference problems. However, this method produces results for patient samples that agree closely with the results from isotope dilution mass spectrometry (ID-MS). This motivated our use of the re-expressed MDRD equations (MDRD-4 and MDRD-6) instead of the original equation [4], because the original MDRD equation was developed for use with ID-MS traceable serum creatinine [30]. Second, ANN models can be difficult to display as equations and cannot be readily used without special software, so physicians may be reluctant to accept the results of ANN models. There is need for a platform that can display ANN models and that allows other researchers to readily perform external validation. Third, a previous study-indicated that GFR estimated by 99mTc-DTPA dynamic renal imaging might not better than the modified abbreviated MDRD equation [31], and the renal dynamic imaging method was less accurate than CKD-EPI equation as well [32]. However, the same study found that the two methods performed similar capability in determining GFR among higher-GFR patients [32], and ^99m^Tc-DTPA dynamic renal dynamic imaging yields accurate results that are nearly the same as those from measurements of inulin clearance [33]. Rehling et al. showed that a regression line between the values measured by these different methods did not differ from the line of identity [33]. Ma et al. (2007) suggested that, using proper reference GFR, more adequate background subtraction, and soft-tissue attenuation correction may improve the accuracy of ^99m^Tc-DTPA dynamic renal imaging [31]. Finally, our RBF network predicted a higher GFR than that from ^99m^Tc- DTPA renal dynamic imaging. This might be due to differences of participants in the training group (CKD patients with and without diabetes [20]) and the study group (diabetes patients with and without normal kidney function). Use of more similar training and study groups would provide better external validation and may provide improved results.

A recent survey in China [34] showed that the prevalence of diabetes was 9.7%, corresponding to 92.4 million people. Although some of the established methods used to estimate GFR are suitable for Chinese patients with CKD [22], it is important to have more accurate and precise estimations of GFR. In some measures of accuracy and precision, our RBF neural network performed significantly better than the re-expressed MDRD equations in the estimation of GFR. In particular, the IQRs (Table 3) and 95% limits of agreement (Figure 1) for the eGFR_RBF_ were smaller than those from eGFR_4_ and eGFR_6_, indicating better precision for the RBF network. However, our data indicated that eGFR estimated by the RBF neural network tended to be higher than the sGFR, and this would result in under-estimation of CKD stage. We suggest that use of an RBF network model with more variables and testing of the model with additional data sets may ultimately provide more accurate and precise estimates of GFR.

## Conclusions

In patients with type-2 diabetes, GFR estimated by our RBF network provided better precision and accuracy for some groups of patients than GFR estimated by the traditional MDRD equations. However, the RBF network estimates of GFR tended to have greater bias and higher than those indicated by sGFR determined by ^99m^Tc-DTPA renal dynamic imaging.

## Competing interests

The author declares that they have no competing interests.

## Authors’ contributions

XL, TQ-L: planning of the project; XL, YR-C CW and ML: carrying out of the experimental work; XL, NS-Li, LS-Lv and XM-W: intellectual analysis of the data; XL: writing of the paper. All authors read and approved the final manuscript.

## Pre-publication history

The pre-publication history for this paper can be accessed here:

http://www.biomedcentral.com/1471-2369/14/181/prepub
